# Analysis of a Large Database of Concrete Core Tests with Emphasis on Within-Structure Variability

**DOI:** 10.3390/ma12121985

**Published:** 2019-06-20

**Authors:** Angelo Masi, Andrea Digrisolo, Giuseppe Santarsiero

**Affiliations:** School of Engineering, University of Basilicata, Viale dell’Ateneo Lucano, 10, 85100 Potenza, Italy; angelo.masi@unibas.it (A.M.); andrea.digrisolo@unibas.it (A.D.)

**Keywords:** existing buildings, reinforced concrete, seismic vulnerability assessment, in situ concrete strength, variability of concrete strength

## Abstract

In reinforced concrete (RC) structures, the compressive strength of concrete can play a crucial role in seismic performance and is usually difficult to estimate. Major seismic codes prescribe that concrete strength must be determined essentially from in situ and laboratory tests. Mean values obtained from such tests are the reference design values when assessing existing structures under seismic actions. The variability of concrete strength can also play an important role, generally requiring that various homogeneous domains are identified in a single structure, in each of which a specific mean value should be assumed as representative. This study analyzes the inter- and intra-variability of the concrete strength of existing buildings using a very large database made up of approximately 1600 core tests extracted from RC buildings located in the Basilicata region (Southern Italy). The analysis highlighted that concrete strength variability was dependent on the structures’ dimensions as well as on the number of storeys. Moreover, the concrete strength of cores extracted from columns was found to be, on average, lower than that from beams, thus justifying the usual practice to extract cores mainly from columns, which results in a conservative approach as well as a more feasible one. Finally, some case studies were analyzed, specifically focusing on the effects of the within-storey variability. Conservative strength values, to be used especially in the case of vertical members subjected to high axial loads, are suggested.

## 1. Introduction

In order to more effectively face natural risks, civil protection activities should be devoted to post-earthquake emergency, and above all, to prevention through a wide range of risk mitigation programs. Focusing in particular on the seismic risk reduction of buildings, vulnerability assessment is of fundamental importance for several reasons, among which: (i) to evaluate current safety conditions in view of possible urgent decisions; (ii) to define priorities and timescales in carrying out extensive strengthening programs; and (iii) to drive towards strengthening interventions that are highly effective in terms of the benefit–cost ratio.

The knowledge of concrete strength is essential to perform seismic and gravity load assessments of older reinforced concrete (RC) buildings. Reinforced concrete buildings, in fact, are frequently not provided with proper reinforcement detailing, thus brittle crises can occur in beam and column (or walls) members, and most importantly, in beam–column joints equipped with either deep beams [1] or wide beams [2,3]. The members’ strength related to brittle failure modes is strongly related to concrete strength which, as a consequence, plays a key role in the assessment path. 

Reliable seismic and gravity load assessments are also important in defining intervention priority, although it should be recognized that these decisions are based also on other socio-political demands and criteria. 

Further, the knowledge of seismic vulnerability at the territorial scale can allow the elaboration of seismic scenarios for the most probable seismic events which can occur in a given area. Regarding this issue, some procedures aimed at large-scale vulnerability assessment of buildings (e.g., [4]) based on typological characteristics, have been developed. As these methods are based on poor data, their level of accuracy is limited. 

In a similar context, other studies (e.g., [5]) evaluated the territorial distribution of seismic risk at the national scale, providing risk maps based on the current Italian hazard map [6]. The structural vulnerability was, in this case, evaluated with a detailed procedure (mechanical methods), based on the material properties reported in References [7,8], where mean and standard deviation values of the concrete strength of RC buildings built before 1970 were provided. The mechanical properties were derived from compression tests on concrete cubes at the Laboratory of Testing Materials of the University of Naples performed in the framework of the routine controls prescribed during the construction of new buildings. However, strength values obtained in such a way do not take into account the expected remarkable differences between current in situ strength (generally many years after the construction) and laboratory test results carried out during the construction.

For this reason, it appears appropriate to refer to the results of compression tests on concrete cores extracted from RC buildings at the time the seismic assessment is being carried out. Indeed, in Italy, starting from 2004, a huge (and still ongoing) assessment program was started to draw a picture of public buildings’ vulnerability in order to allow owners and political authorities to make decisions and time scaling in view of an extensive strengthening plan. In fact, once the results of the seismic assessment are available, decisions specifically applicable to single buildings can be taken. First of all, the convenience of strengthening (either retrofitting or upgrading) with respect to replacing should be evaluated, and secondly, a comparison with other buildings should be made in order to establish priorities [9].

As a result of more than ten years of seismic assessments of public buildings, a large amount of data on concrete strength values obtained from a laboratory compression test on cores extracted from these buildings are available. As an example, Ferrini et al. [10] obtained data regarding approximately 1500 concrete cores extracted from 400 buildings located in the territory of the Tuscany region. 

A similar database on concrete core tests (approximately 1600 specimens) is described and analysed in the present paper. They were collected as a result of the seismic assessments conducted in the Basilicata region on RC buildings whose construction period ranges between 1940 and 1990. Therefore, concrete strength distribution depending on the construction period can be determined. First, this can provide a useful tool for seismic vulnerability assessments at the regional level [11]. Secondly, the knowledge of mean strength values can be helpful, although not sufficient, in the structural evaluation of single buildings. In fact, in this case, the variability of concrete strength also matters, and it is quite important to identify homogenous areas over a single building, that is areas where a single value (generally the mean value according to seismic code provisions), can be assumed as representative. Some studies showed that the concrete strength variability inside a single building can be rather large, up to values of the coefficient of variation (CV) equal to 0.50 [12,13]. Other researchers showed that the CV values usually decrease when the strength increases [14].

In Reference [15], the high variability of the concrete strength, even along a single structural member, is discussed. The CV values ranging between 14% and 31% in different areas of the investigated member are found. Reasons for this variability and suggestions on how to manage it are provided in this paper. In Reference [16], a method to assess the variability of concrete strength along a single member using a rebound hammer test and compression tests on cores is proposed. 

Accounting for the frequent large variability of concrete strength within a single building, a procedure for identifying homogenous areas is proposed in Reference [17], based on a first investigation phase made up of in situ, non-destructive tests. The results of these tests allow the definition of an effective, although as limited as possible, campaign of core drilling. Other studies provide general empirical expressions and approaches [18,19] estimating the in situ concrete strength variability using non-destructive test (NDT) results.

In the present paper, an updated version of the database reported in Reference [11] is studied and further analyses are made mainly aimed at providing indications on the within-building concrete strength variability, especially focusing on the influence of building size (particularly, the number of storeys) on CV values. Additionally, some remarks are also made by comparing strength values related to cores extracted from either beam or column members. Finally, some suggestions on the determination of the design strength to be used in the safety verifications prescribed by structural codes on existing buildings, under seismic loads, are proposed.

## 2. Description of the Core Tests’ Database 

Data analyzed in this study were collected within a large program of seismic vulnerability assessments of public buildings located in the Basilicata region (Southern Italy), which was managed by the local regional authorities (i.e., local governments), as prescribed by the national law OPCM 3274/2003 [20]. The assessment program involved strategic buildings, such as hospitals, and buildings at significant risk of collapse, such as schools. In this framework, a series of compression test results on concrete cores drilled from the buildings under evaluation was obtained. Therefore, the structural members (beam, column or wall) and the location where core specimens were extracted was the choice of the technician in charge of the specific evaluation. Results in terms of concrete compression strength were reported in the test certificates also providing other information regarding the core specimens (location in each structure, height, diameter, specific weight, etc.). The database is made up of 1572 concrete cores extracted from 346 structures, 280 of which are school buildings and 66 which are hospital buildings. Globally, 968 cores were extracted from schools (on average 3–4 cores/building) and 604 cores from hospitals (on average 9 cores/building).

In order to correct the strength values directly achieved on core specimens, possibly different from in situ strength, thus obtaining comparable results, the core strength values f_core_ were converted into the corresponding in situ strength f_c_ through the Relationship (1) reported in Reference [15].

(1) $$f_{c}=\left(C_{H/D}\cdot C_{\mathrm{dia}}\cdot C_{\mathrm{st}}\cdot C_{d}\right)\cdot f_{\mathrm{core}}$$

where C_H/D_ is the correction coefficient for the height/diameter ratio H/D different from 2.0; C_dia_ is the correction coefficient for the diameter of the core; C_st_ is the correction coefficient for the presence of reinforcing bars; and C_d_ is the correction coefficient for damage due to the drilling. Regarding this latter factor C_d_, FEMA 274 [21] suggests assuming a constant value C_d_ = 1.06, while other researchers [22] suggest assuming 1.20 for f_core_ < 20 MPa, and 1.10 for f_core_ > 20 MPa, considering that the lower the strength, the higher the expected drilling damage. Following this approach, in the present paper, the values reported in Reference [23] have been used, assuming:

- C_d_ = 1.30 for f_core_ ≤ 10 MPa;
- C_d_ = 1.20 if 10 < f_core_ ≤ 20 MPa;
- C_d_ = 1.10 if 20 < f_core_ ≤ 30 MPa;
- C_d_ = 1.00 if f_core_ > 30 MPa.

In the following, some basic analyses on the database in terms of f_c_ values are reported.

Data was first analyzed in respect to the construction period of buildings from which cores were extracted. Four construction periods, being related to the enforcement of important structural codes on an RC buildings in Italy, were considered, namely, <1961, 1961–1971, 1972–1981, and >1981.

Table 1 reports the number of cores and buildings for each construction period with the related mean and median values of in situ strength fc, as well as the related dispersion in terms of standard deviation and coefficient of variation CV.

**Figure 1:**
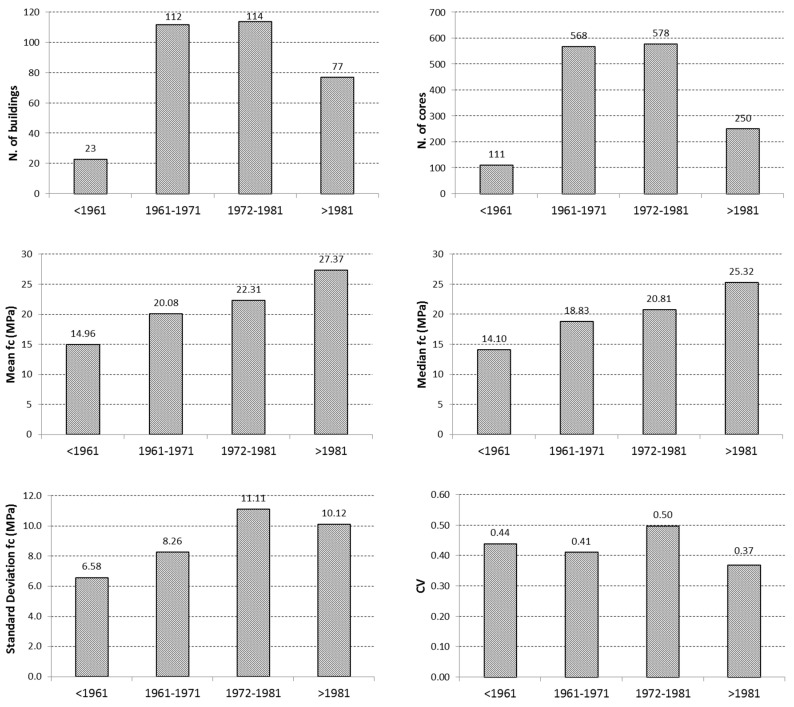
Distribution of the main statistical data related to the core strength values as a function of the construction period.

Data reported in Table 1 are also displayed in the graphs in Figure 1. As can be seen, most of the buildings, and consequently the cores, belong to the period 1961–1981, i.e., the years of the maximum economic growth in Italy and also in the Basilicata region. As expected, both the mean and median values of concrete strength increased with time, possibly due to the enforcement of building codes providing more severe rules on the control of materials’ quality during the construction of new buildings.

Vice versa, the standard deviation and coefficient of variation values did not show a well-defined trend over time. However, as can be noted, the dispersion was generally high with the CV values being in the range of 0.37–0.50.

**Figure 2:**
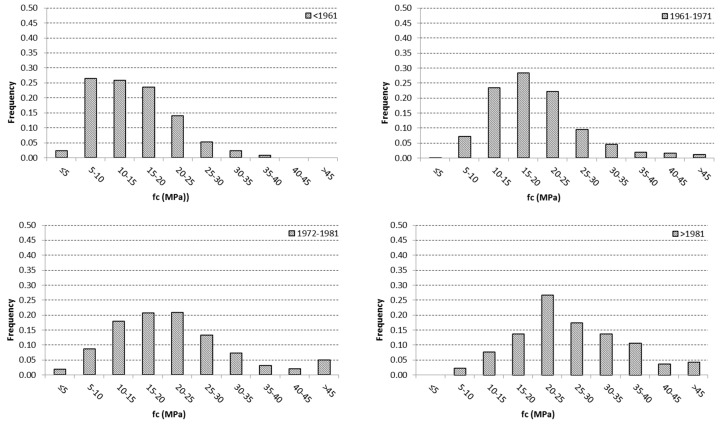
Distribution of the f_c_ values in the construction periods under consideration.

In Figure 2, the distributions of f_c_ values in the four construction periods are displayed, confirming the high dispersion, especially for the period 1972–1981. It is worth noting that also for the more recent periods (i.e., 1972–1981 and >1981) there was a significant quota of buildings with very low concrete strength (<10 MPa). Moreover, the last two periods did not show an improvement in terms of concrete homogeneity, as could be expected after the enforcement of a new code on RC constructions.

**Figure 3:**
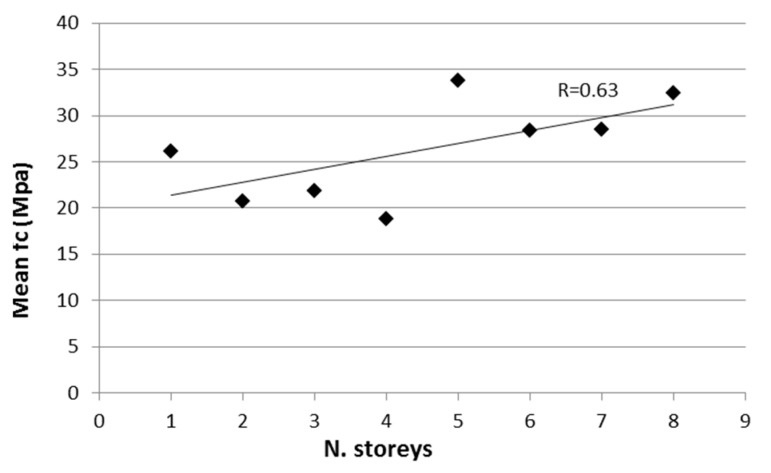
Mean concrete strength as a function of the number of storeys.

Figure 3 displays the variability of the mean concrete strength as a function of the number of storeys. For each group of buildings with a given number of storeys, the mean concrete strength was computed, discarding buildings with fewer than five cores. As a result, the database is made up of 100 building. As can be seen, concrete strength had a clear trend of increasing with the number of storeys. It can also be noted that an evident difference was visible between buildings with four storeys or less and buildings with five to eight storeys. This could be due to the better concrete quality, workmanship, and supervision adopted for the construction of larger buildings.

## 3. Analysis of Strength Values by Structural Member Type

When planning a destructive tests campaign, a crucial issue relates to the choice of the sampling points. Current seismic codes on the evaluation of RC buildings provide rules forcing the extraction of cores from both column and beam members. However, due to the practical constraints, difficulties in extracting cores from beams are well known among structural engineers, and more importantly, in some cases, extraction can be almost impossible (e.g., flat or wide beams having the same thickness as the adjacent floor slab). Moreover, even in the case of deep beams, firmly fixing the drilling machine could be rather difficult. As a result, in most cases, core drilling is carried out on vertical members (column and walls), and only in a few cases on beams.

This is clearly revealed by the database under study, where most cores were extracted from columns, that is approximately 1400 out of a total of ~1600 cores.

**Figure 4:**
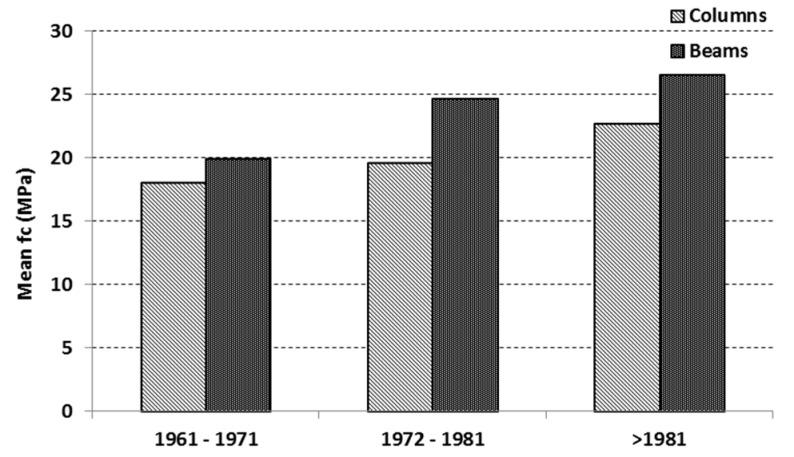
Mean strength values of cores extracted from columns and beams (computed by averaging, in each period, the mean values of the core strength from columns and beams relevant to each single building).

In order to understand if/how the strength of the cores drilled from columns can be assumed as representative also of beams and then used to determine the design value to be used in safety verifications, the database was analyzed to highlight possible differences between column and beam cores (Table 2). The comparison was made considering only the buildings where cores extracted from both columns and beams were available and assuming that the concrete used in the column members had the same mix design as that used in the beams, as it is a common construction practice. Therefore, the total number of values to be compared decreased to 240 (156 cores from columns and 73 from beams, extracted from 41 buildings). The comparison is displayed in Figure 4 as a function of different construction periods, where the period <1961 was not considered due to the low number of available values. Note that the mean values reported in Figure 4 were computed by averaging, in each period, the mean values of core strength (separately for columns and beams) related to each single building.

As displayed in Figure 4, for all construction periods, the mean strength of cores extracted from beams was always higher than that from columns, with a difference in the range 1.10–1.25.

In order to evaluate whether the observed differences were significant, Students’ *t*-tests were performed on the two strength value sets (columns versus beams). The tests can be applied in two different ways:

- two-tailed test: to know whether the mean values are equal;

- one-tailed test: to know whether a mean is higher than the other.

If one cannot exclude that a mean value is higher than the other, the two-tailed test is preferable. 

The *t*-test assumes a null hypothesis (H_0_), meaning that the mean related to the two sets values (μ_C_ for columns, μ_B_ for beams) belong to the same population, the differences being due to the random variations, and the alternative hypothesis (H_1_) that the two mean values belong to different populations, that is:

H_0_: μ_C_ = μ_B_ (2)

H_1_: μ_C_ ≠ μ_B_ (3)

The test was carried out referring to two significance levels: α = 0.05 and α = 0.01.

Table 3 reports the statistics result T and the limit values for the two significance levels: T0.05 and T0.01. In the last two rows, the result of the *t*-test for each construction period is reported, where OK means that the hypothesis H_0_ cannot be rejected.

For a significance level α = 0.05, the *t*-test yielded a positive result for two out of the three construction periods, while for a significance level α = 0.01, a positive result was found for all the construction periods. Therefore, the differences between the two populations were negligible, and consequently, the strength values of columns and beams can be considered as belonging to the same population. 

Further, there were two additional aspects to be addressed in order to perform core drilling on column members: i) core drilling from columns is generally less expensive and time-consuming; ii) columns play a more important role in the load-bearing structural system under both vertical and seismic loads.

In sum, although the above findings should be considered valid with respect to the database under study, they are nevertheless important as performing core drilling only on columns appears to be on the safe side, with their concrete strength being, on average, lower than that of beams, although they belong to the same population. Therefore, the strength values found on columns’ cores can also be representative of beams’ strength. This remark is consistent with the work in progress to update the Eurocode 8 (EC8)-Part 3: Assessment and retrofitting of buildings and bridges [24], where the recommended minimum requirements for different levels of testing no longer prescribe that cores need to be extracted from each type of primary element (e.g., column and beam), as is prescribed in the current version of EC8-3. Moreover, this also affects the shear strength of beam–column joints since they are cast along with beams. Due to this, they have to be assumed to have the same concrete strength.

## 4. Analysis of Within-building Variability

Previous analyses were devoted to outlining the general features of the whole database under consideration. However, the concrete strength properties can show high variability in the same building, between different storeys, and even at the same storey, although it can be assumed that the concrete used at different storeys has the same mix design, as it is in usual construction practice.

In order to analyze the within-building variability, further analyses were performed only on buildings where a large number of cores were available. Assuming that this number is not less than 5, the new database includes 100 buildings and 802 cores (Table 4). 

**Figure 5:**
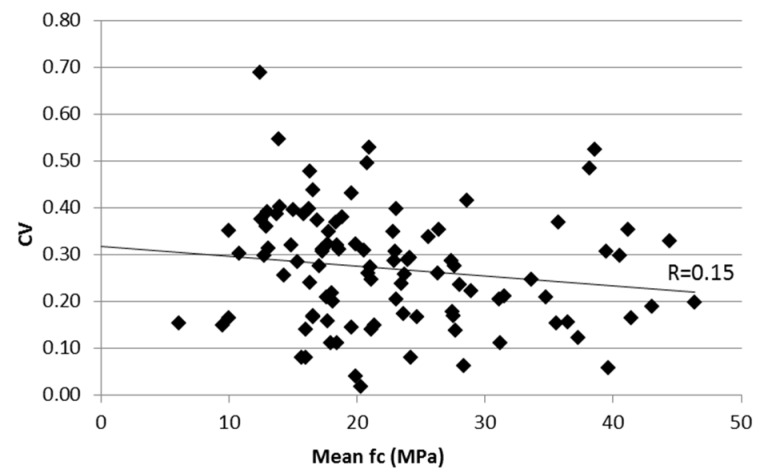
Comparison of CV and mean values computed in each building.

Relating the coefficient of variation to the mean value of the concrete strength in each building (Figure 5), no correlation was found, as already reported in other studies [14,25]. This reveals that an increase in the average quality of concrete in existing buildings is generally not accompanied by a correspondent decrease in the concrete strength variability.

The estimation of concrete strength for design and assessment purposes is related to the choice of the knowledge level (KL) of the structure under study. Three KLs are defined (limited, normal, and full knowledge) in order to choose the appropriate confidence factor (CF) value to be adopted to reduce the concrete strength in the evaluation process. Once a knowledge level is assumed (KL1, KL2 or KL3), the current European and Italian seismic codes define the number of cores to be extracted depending on the total storey area, implicitly assuming that a single storey can be considered a homogeneous area. This assumption appears justifiable given that, except in the case of a very large floor area, the members of a single storey are generally cast almost simultaneously, thus similar concrete properties should be expected. On the other hand, different storeys can be cast after a period of time, leading to possible differences, for example, due to the modified environmental curing conditions (temperature, moisture). This supports the preliminary assumption that different storeys might represent different homogeneous areas. 

**Figure 6:**
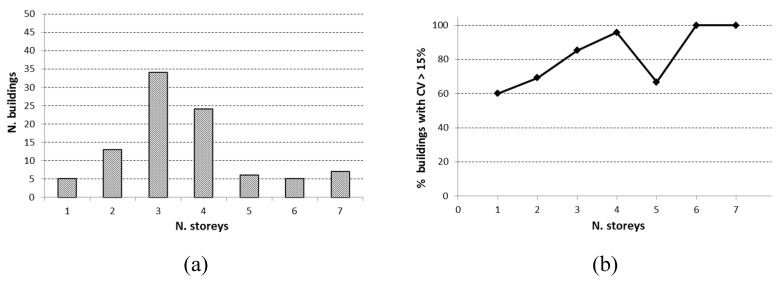
(**a**) Distribution of buildings by number of storeys; (**b**) number of buildings with CV higher than 15%.

On the basis of the above remarks, it is expected that the global dispersion within a whole building should increase with the number of storeys. In fact, Figure 6b displays that, in general, the percentage of buildings where the CV value is higher than 15% (a typical reference value given in the literature and in some structural codes) increases with the number of storeys, with the only exception being the five-storey group, where two buildings out of six have CV < 15%.

Further, the analysis of data showed that the average floor area did not vary significantly with the number of storeys, so the variation of CV values, shown in Figure 6b, was essentially determined by the number of storeys.

This result confirms that considering a whole building as a single homogenous domain can be inappropriate in most cases, as the assumed mean strength value would be associated with an unacceptable high variability.

**Figure 7:**
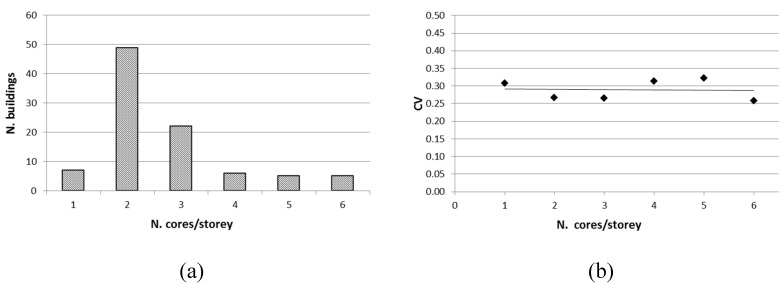
(**a**) Distribution of buildings as a function of the core number extracted from each storey; (**b**) CV values in relation to the mean number of cores extracted by single storeys.

As can be seen in Figure 7, increasing the number of cores extracted from each storey in a building generally did not improve the achieved results in terms of a decrease in dispersion (i.e., CV value does not decrease), similar to the findings in Reference [26], and in contrast to the approach of FEMA 356 [27], which suggests increasing the number of cores in order to reduce the variability. This confirms that different storeys are likely to be homogeneous areas different from each other, and moreover, the variability of concrete strength is often intrinsic to RC buildings.

**Figure 8:**
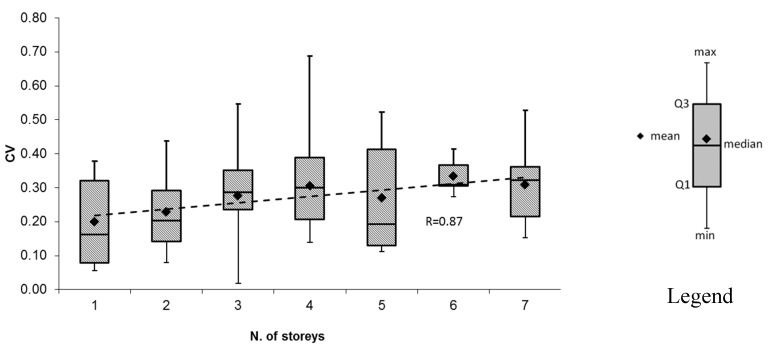
Box-plot of statistical data with respect to the number of storeys.

Looking at the graph in Figure 8, a general increase in the dispersion can be observed when the number of storeys increases. In fact, the mean value of CV is higher for taller buildings, as demonstrated by the trend of the mean values of CV (dotted line in Figure 8). The median value had the same trend, while the standard deviation appeared to be independent of the number of storeys. Indeed, the results showed that even for one-storey buildings, CV was higher than 15%, being around 20%. This result suggests that one-storey buildings could also be made of more than one homogenous area due to the size of the building in plan. In fact, when the size increases, the concrete is likely to belong to more than one batch.

**Figure 9:**
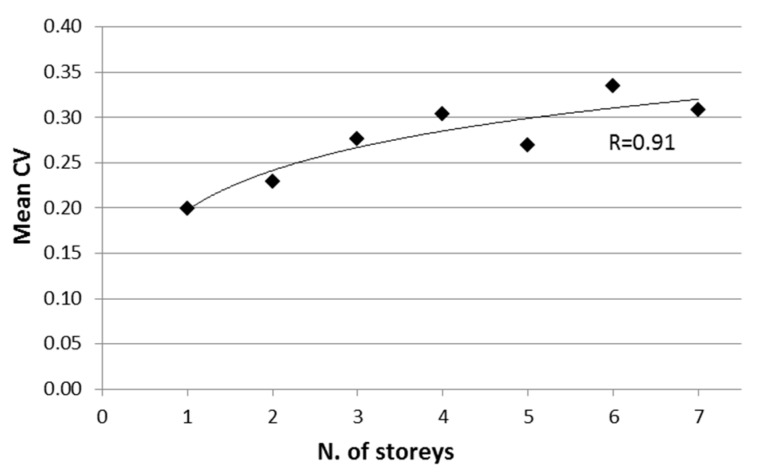
Logarithmic regression between mean values of CV and number of storeys.

The mean CV value correlates well with the number of storeys, as can be seen in Figure 9, where a high correlation factor (*R* = 0.91) was found using the following logarithmic regression relationship:

(4) $$\mathrm{CV}=0.0628\ln\left(n_{p}\right)+0.1979$$

where n_p_ is the number of storeys.

This finding confirms the importance of appropriately identifying the different homogeneous areas in each building accounting for the fact that, in general, the variability of concrete properties increases with the building dimensions, especially with the number of storeys. Further, this finding suggests taking into account the average expected dispersion of concrete strength as a function of the number of storeys when making vulnerability assessments at the territorial scale (i.e., when the seismic vulnerability needs to be assigned to a single or a class of buildings, necessarily assuming a single strength value for each building).

### Analysis of Within-Storey Variability

To further develop the analyses carried out in the previous sections by examining also the within-storey variability, five buildings with a number of storeys ranging from 3 to 7 and with at least 4 cores per storey, were selected (Table 5).

**Figure 10:**
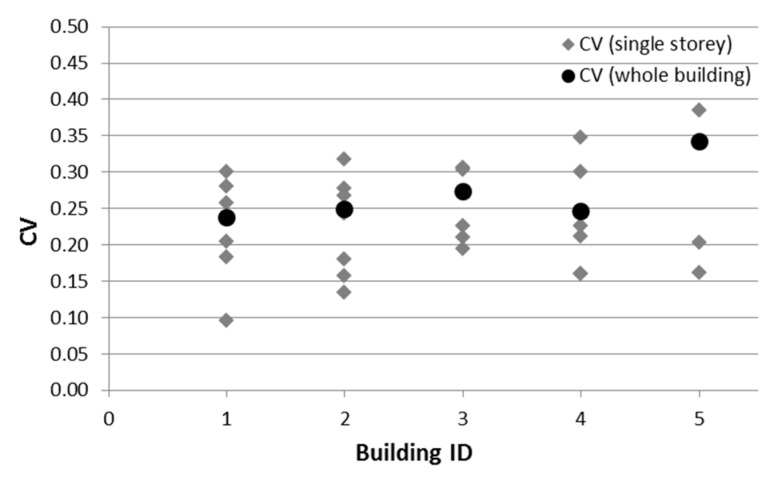
CV values computed in the single storeys and in the whole building.

Figure 10 shows the distribution of the CV values relevant to the whole building (black circles) and to each storey (grey diamond-shaped dots). Firstly, there is a high variability of concrete strength, with the CV values being higher than 0.15 most of the time (although at least four cores per storey were extracted), as shown in the fourth column of Table 5 that reports the number of storeys with a CV < 0.15 out of the total number of storeys for each building. Moreover, it is worth noting that the CV value computed for the whole building was quite different from the CV values relevant to each storey. Once again, this confirms the need to identify homogenous areas in building structures in order to obtain mean values of concrete strength that are adequately representative of each area. On this issue, a broad discussion with remarks and suggestions on a possible approach to be followed is reported in Reference [17].

Previous analyses demonstrated the high variability of concrete strength in a single building and even within a single storey, in most cases, is due to the intrinsic variability of the material properties. Increasing the number of cores did not reduce the dispersion of strength values. This latter result was evidenced by the high number of buildings having a CV larger than 0.15. One should derive that a single strength value cannot be representative of more than one storey, which represents the maximum extent of the so-called homogenous areas. Indeed, in some cases, more homogenous areas should be set for a single storey, so that any possible irregularity in the plan due to the concrete strength variability [28,29] is not neglected. Moreover, also in the case of seismic evaluation, dealing with high within-storey variability using the mean value could be not on the safe side due to the different role of concrete strength in determining the capacity of structural members (e.g., ductile versus fragile members).

**Figure 11:**
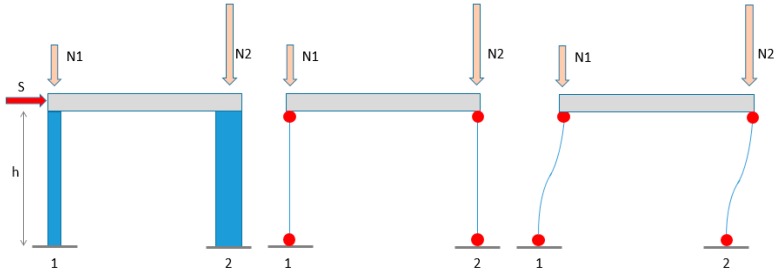
Example portal frame.

A lower strength value on the larger structural members (especially in the case of columns) can lead to remarkable negative effects on the total structural capacity. This is especially true for frame structures with high axial load values on the columns. In order to clarify this aspect, a simple example was considered: a shear-type portal frame made up of two columns with dimensions of 300 × 300 mm for column n.1 and 300 × 600 for column n.2 (see Figure 11), provided with an amount of longitudinal reinforcement of 0.5% (typical of pre-code buildings), meaning A_s1_ = 450 mm^2^ for column n.1 (four 12 mm diameter bars) and A_s2_ = 900 mm^2^ for column 2 (six 14 mm diameter bars). The storey height was set to h = 3000 mm.

As for concrete strength, the characteristics of the 2nd storey of building no.1 (Table 5) were considered, provided with a mean concrete strength f_med_ = 19.53 MPa and minimum strength f_min_ =10.68 MPa. The steel yielding stress was assumed as f_y_ = 320 MPa.

Axial load values proportional to the columns’ gross area were assumed (as in typical design practice), i.e., equal to 40% of the ultimate axial load determined as N_u_ = A_c_·f_min_. This means that the axial load value on column 1 was about N_1_ = 360 kN, while that on column 2 was N_2_ = 720 kN.

Based on the assumption of shear type, a double plastic hinge is expected to develop at column ends in a storey failure mechanism due to the horizontal actions. So, the frame base shear is directly proportional to the yielding moments developed at column ends. The total horizontal strength capacity of the structure is then:

(5) $$S=\frac{2\cdot M_{y1}+2\cdot M_{y2}}{h}$$

Yielding moment values for the two columns using either f_med_ (case “a”) or f_min_ (case “b”) can be computed:

(a) M_y1a_ = 60.0 kNm, M_y2a_ = 238.2 kNm
(b) M_y1b_ = 50.6 kNm, M_y2b_ = 185.6 kNm

Now, two scenarios are assumed:

1. Column 1 having concrete strength f_med_ and column 2 f_min_
2. Column 1 having concrete strength f_min_ and column 2 f_med_

In scenario 1, the total strength capacity is:

(6) $$S_{1}=\frac{2\cdot M_{y1a}+2\cdot M_{y2b}}{h}=163.7\;\mathrm{kN}$$

While in scenario 2:

(7) $$S_{2}=\frac{\cdot 2\cdot M_{y1b}+2\cdot M_{y2a}}{h}=192.5\;\mathrm{kN}$$

As can be seen, scenario 1 results in a total base shear 15% lower than scenario 2.

This means that assuming a strength value equal to the mean concrete strength can lead to overestimation of the storey shear capacity due to the possible presence of low strength values on structural members such as larger columns or walls. This happens even for ductile mechanisms such as flexure for columns, for which the concrete strength plays an important role in the presence of significant axial load values. In order to visualize this situation, the base shear values of the example portal frame in scenarios 1 and 2 were plotted as a function of the axial load. 

**Figure 12:**
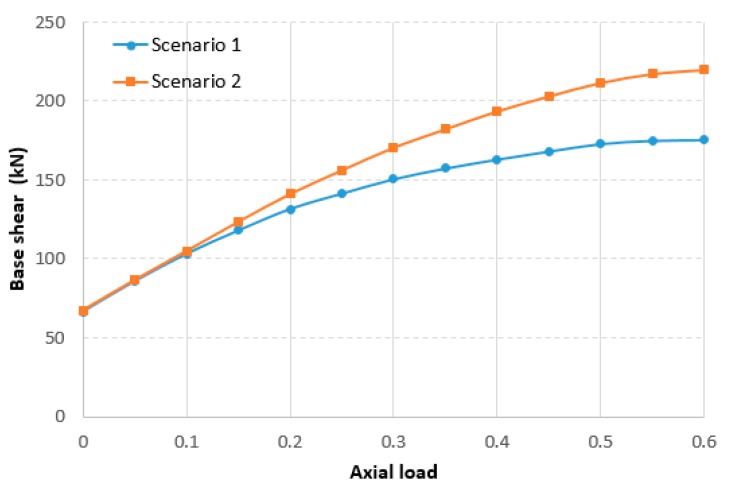
Base shear as a function of the normalized axial load.

As can be seen from Figure 12, resulting base shear values of the portal with inverted concrete strength values (scenarios 1 and 2) diverge when axial load increases. In this regard, it should be taken into account that existing RC buildings, designed only according to gravity loads, often have high axial load values as a result of the non-seismic design approach.

Therefore, for storeys with high concrete strength variability (CV > 0.15), it is advisable to use the mean value minus one standard deviation, as proposed by FEMA 356 [27], in order to obtain more conservative results. However, it should be noted that seismic codes frequently allow the collection of an insufficient number of cores to determine CV and standard deviation. In fact, for small–to medium-sized residential buildings, at most, three cores per storey can be drilled (KL3), irrespective of the floor surface.

Therefore, it is strongly suggested to integrate destructive tests with non-destructive tests in order to obtain a sufficient number of strength values [17]. However, in situ material tests give rise to remarkable direct costs (drilling and non-destructive testing operations with related repair works) and indirect costs (related to occupancy disruption). Due to these issues, concrete strength values alternative to the mean minus one standard deviation should be assumed in cases where few strength values are available.

**Figure 13:**
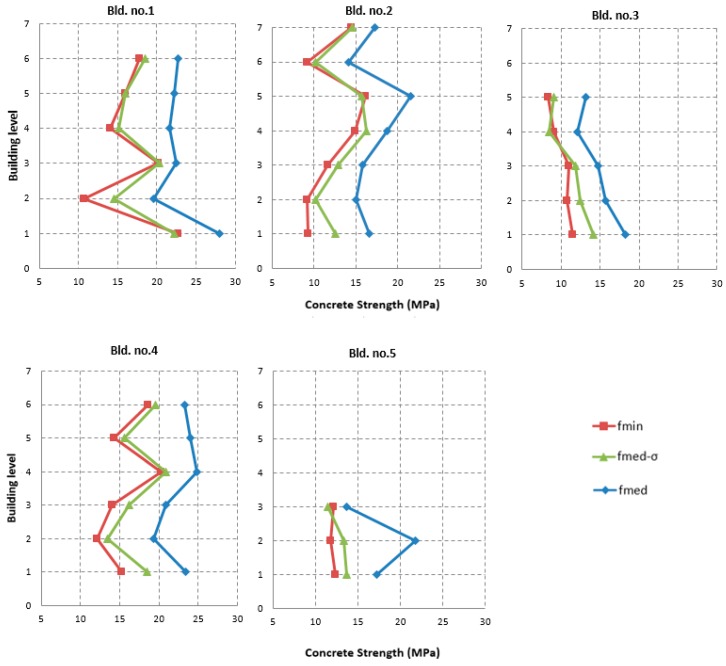
Comparison of the strength values at different heights for the five selected buildings.

In order to derive some indication, the distributions of concrete strength along the height of the five selected buildings are plotted in Figure 13. In particular, for each storey, the mean value f_med_, the minimum f_min_, and f_med–σ_ are displayed.

As can be seen from Figure 13, f_med–σ_ is generally very close to f_min_. For example, in buildings no. 1, 2, and 4, the two strength values had almost the same trend, except for the 1st storey of buildings no. 2 and 4 and the 2nd storey of building no. 1. Buildings no. 3 and 5 showed larger differences at the first storeys, although a good correspondence between f_min_ and f_med–σ_ was generally found.

Based on the above considerations, in the case of seismic assessment where concrete strength was based on few cores per floor, and high scatter was observed among related strength values; it seems advisable to use the minimum concrete strength value of each floor in place of the mean. In fact, the results showed that the minimum value was somehow representative of f_med–σ_, that is, a more conservative strength value is suggested by FEMA356 [27] when dispersion is high.

## 5. Concluding Remarks 

The availability of a large database of seismic assessments of RC buildings located in Basilicata (Italy) allowed the analysis of concrete strength properties (evaluated by means of core tests), in terms of mean values and variability, across different construction periods. First, the analyses showed that the concrete strength in column members was, on average, lower than that in beams. This outcome has important implications in practical seismic assessments of existing RC buildings, justifying the extraction of concrete cores only from columns, being both on the safe side and more feasible. Further, determining the concrete strength directly on columns appears still more remarkable accounting for their crucial role in carrying gravity loads.

After these preliminary analyses, the within-building variability of concrete strength was investigated. It was found that the coefficient of variation of concrete strength was mostly higher than the limit value provided by structural codes (e.g., Italian code), and was almost independent of the number of cores extracted from each storey. Looking at large-scale vulnerability assessments, where mean values as well as variability of concrete strength are required, a logarithmic relationship between CV and the number of storeys was found. 

The variability of concrete strength was specifically analyzed in five buildings where a large number of cores was available (i.e., at least four cores per storey). The results showed that the variability across the whole building was comparable to that relevant to each single storey. 

Analyzing the within-storey variability of concrete strength, it was found that using the mean value in the presence of a high dispersion of strength values can lead to an overestimation of the storey capacity, especially when lower strength values were related to the larger structural member such as large columns or shear walls. The influence of axial load values in this regard was also examined, underlining that this also happens in the presence of ductile mechanisms, such as flexure in column members. In order to overcome this, it is suggested to assume as a design value the mean value minus one standard deviation, provided that a sufficient number of test values are available for each floor. Taking into account that in usual practice, due to the costs and occupancy disruption constraints, the number of in situ tests at each storey is generally less than three, and it is not possible to calculate dispersion parameters (CV and standard deviation). In these cases, particularly in the case of significant differences among the available strength values, it is suggested to assume the minimum value from tests as the design value.

## Figures and Tables

**Table 1:** Main statistical values of the available data on concrete strength f_c_.

Construction Period	<1961	1961–1971	1972–1981	>1981	ND ^1^
Number of buildings	23	112	114	77	20
Number of cores	111	568	578	250	65
Mean value (MPa)	14.96	20.08	22.31	27.37	23.47
Median value (MPa)	14.10	18.83	20.81	25.32	22.77
Standard deviation (MPa)	6.58	8.26	11.11	10.12	7.81
Coefficient of variation	0.44	0.41	0.50	0.37	0.33

^1^ ND: not defined.

**Table 2:** Statistical data of the subset of concrete strength values regarding buildings with cores extracted from beams and columns (B = beams, C = columns).

Construction Period	<1961	1961–1971	1972–1981	>1981
Number of buildings	2	15	11	13
Number of cores (B)	8	31	23	22
Number of cores (C)	16	56	44	40
Mean value (MPa) (B)	23.8	19.9	24.7	26.6
Mean value (MPa) (C)	17.2	18.0	19.6	22.7
Standard deviation (MPa) (B)	7.20	5.89	9.88	5.49
Standard deviation (MPa) (C)	2.61	5.38	7.80	4.69
Coefficient of variation (B)	0.30	0.30	0.40	0.21
Coefficient of variation (C)	0.15	0.30	0.40	0.21

**Table 3:** The *t*-test on concrete strength values extracted from vertical members and beams.

Construction Period	1961–1971	1972–1981	>1981
T	−1.53	−2.29	−2.90
T_0.05_	2.28	2.29	2.30
T_0.01_	2.88	2.91	2.91
R_0.05_	OK	OK	NO
R_0.01_	OK	OK	OK

**Table 4:** Statistical data of the subset of concrete strength values regarding buildings with at least five cores.

Construction Period	<1961	1961–1971	1972–1981	>1981	ND
Number of buildings	8	33	40	16	3
Number of cores	69	257	339	105	32
Mean value (Mpa)	17.45	20.78	22.51	30.18	21.60
Median value (Mpa)	18.40	18.65	20.39	29.43	18.45
Standard deviation (Mpa)	4.46	7.12	9.92	8.06	5.85
Coefficient of variation	0.26	0.34	0.44	0.27	0.27

**Table 5:** Selected buildings for the analysis of within-storey variability.

Building ID	Construction Period	No. of Storeys	No. of Storeys with CV < 0.15	No. of Cores (Total)	No. of Core/Storey	No. of Core/Storey (min–max)	CV (Total) ^1^	CV Storey (min–max)
1	1961–1971	6	1/6	33	5.5	5–6	0.24	0.10–0.30
2	1961–1971	7	1/7	63	9.0	6–12	0.25	0.13–0.32
3	<1961	5	0/5	34	6.8	6–8	0.27	0.20–0.31
4	1972–1981	6	0/6	56	9.3	6–13	0.25	0.16–0.35
5	1961–1971	3	0/3	17	5.7	5–6	0.34	0.16–0.39
	Total	27	2/27	203	7.5			

^1^ referred to whole building.
